# CalR is an activator of biofilm formation in *Vibrio parahaemolyticus*

**DOI:** 10.1128/aem.00724-25

**Published:** 2025-09-11

**Authors:** Jingyang Chang, Yining Zhou, Miaomiao Zhang, Xue Li, Nan Zhang, Xi Luo, Bin Ni, Renfei Lu, Yiquan Zhang

**Affiliations:** 1 Department of Clinical Laboratory, Nantong Third People’s Hospital, Afﬁliated Nantong Hospital 3 of Nantong University 66479, Nantong, Jiangsu, China; 2 Department of Laboratory Medicine, School of Medicine, Jiangsu University 12676, Zhenjiang, Jiangsu, China; Anses, Maisons-Alfort Laboratory for Food Safety, Maisons-Alfort, France

**Keywords:** *Vibrio parahaemolyticus*, biofilm formation, CalR, regulation, c-di-GMP

## Abstract

*Vibrio parahaemolyticus*, the leading cause of seafood-associated gastroenteritis, often exists in a biofilm state. Biofilm formation in this bacterium is regulated by a complex network of factors, including the second messenger c-di-GMP. CalR, a LysR-type transcriptional regulator, has been demonstrated to play a role in regulating the expression of virulence genes and swarming motility of *V. parahaemolyticus*. In this study, we demonstrate that CalR positively regulates biofilm formation by enhancing the production of exopolysaccharides (EPSs), extracellular proteins, extracellular DNA (eDNA), and c-di-GMP. RNA sequencing reveals that CalR regulates the transcription of 167 genes, including those potentially involved in c-di-GMP metabolism (e.g., *vpa0184*, *vpa0198*, and *vpa1429*) and biofilm-associated regulators (e.g., *scrP*). CalR directly suppresses the transcription of *scrP*, *vpa0184*, and *vpa0198* while activating *vpa1429* transcription in a direct manner. Additionally, CalR directly activates the transcription of *cpsA* but indirectly activates that of *scvE*, both of which are involved in EPS production. In summary, these findings elucidate the regulatory mechanisms by which CalR promotes biofilm formation in *V. parahaemolyticus* and highlight its role in modulating c-di-GMP homeostasis and biofilm matrix production. This work enhances our understanding of the regulatory network governing biofilm formation by *V. parahaemolyticus*.

**IMPORTANCE:**

Biofilm formation is a critical survival strategy for *V. parahaemolyticus*, allowing it to persist in adverse environments. Understanding the regulatory mechanisms underlying biofilm formation is essential for developing strategies to control infections caused by this pathogen. This work demonstrates that CalR positively regulates the production of EPS, extracellular proteins, and eDNA, as well as biofilm formation by *V. parahaemolyticus*. CalR regulates the global gene expression in *V. parahaemolyticus*, including genes such as *scrP*, *vpa0184*, *vpa0198*, and *vpa1429*. ScrP negatively impacts *V. parahaemolyticus* biofilm formation, whereas *vpa0184*, *vpa0198*, and *vpa1429* contribute to c-di-GMP metabolism. CalR represses *scrP*, *vpa0184*, and *vpa0198* while activating *vpa1429* and also promotes the production of c-di-GMP. These findings indicate how CalR positively regulates biofilm formation by *V. parahaemolyticus*.

## INTRODUCTION

*Vibrio parahaemolyticus* naturally inhabits marine ecosystems and can cause illness through consuming contaminated seafood or, less commonly, via exposure of small open wounds to seawater (1). Pathogenic isolates of *V. parahaemolyticus* typically produce multiple virulence factors that are closely associated with their pathogenicity. These factors include thermostable direct hemolysin (TDH), TDH-related hemolysin (TRH), type III secretion systems (T3SS1 and T3SS2), type VI secretion systems (T6SS1 and T6SS2), capsular polysaccharide (CPS), and extracellular proteases (2–4). These virulence factors serve diverse physiological functions and play distinct roles in the pathogenicity of *V. parahaemolyticus*.

*V. parahaemolyticus* can form biofilms, which are matrix-enclosed bacterial communities on surfaces and are widely used by bacterial species for environmental fitness and transmission (5). The biofilm matrix, comprising over 90% of a biofilm, consists primarily of exopolysaccharides (EPS), extracellular proteins, extracellular DNA (eDNA), lipids, and water (6). EPS production in *V. parahaemolyticus* is associated with the gene loci *cpsA-J* and *scvA-O* (7). Deletion of any of these genes significantly reduces biofilm formation (7). The *cps* locus (not *scv*) directly governs the transition between wrinkled and smooth colony morphologies, with wrinkled spreaders producing more EPS (7, 8). However, biofilm formation by *V. parahaemolyticus* correlates strongly with eDNA and extracellular protein content, but not with EPS levels (9). Additional structures such as flagella and CPS also influence biofilm formation (5, 10). Flagella-mediated motility enables bacteria to overcome surface tension and reach surfaces, promoting biofilm formation while also allowing detachment from biofilms to resume planktonic lifestyles (5). *V. parahaemolyticus* possesses polar and lateral flagellar systems: the polar flagellum drives swimming motility in liquids, whereas lateral flagella facilitate swarming motility on surfaces (11). Both motility types are linked to biofilm formation (5, 12). CPS, a mucoid layer covering the cell wall, is negatively correlated with biofilm formation (13). High CPS expression results in opaque (Op) colony morphology, while low or absent CPS expression produces translucent (Tr) colonies (14, 15). *V. parahaemolyticus* can reversibly switch between Op and Tr states (Op-Tr phase variation) under specific conditions (15).

Bis-(3′−5′)-cyclic dimeric GMP (c-di-GMP) is a ubiquitous second messenger in bacteria that controls multiple reactions and bacterial behaviors (16). Its biosynthesis is catalyzed by the GGDEF domain of diguanylate cyclase (DGC), while its degradation is mediated by the EAL or HD-GYP domain of phosphodiesterase (PDE) (16). *V. parahaemolyticus* encodes 62 proteins potentially associated with c-di-GMP metabolism, including 28 with GGDEF domains, 13 with EAL domains, 16 containing both GGDEF and EAL domains, and 5 with HD-GYP domains (17). Among these, the PDEs ScrC, ScrG, GepA (all dual GGDEF/EAL domains), and TpdA (EAL domain) degrade c-di-GMP, inhibiting biofilm formation and activating flagellar motility (18–21). Similarly, VopY, another EAL domain-containing protein, enhances T3SS2-dependent enterotoxicity and cytotoxicity by reducing c-di-GMP levels (22). Conversely, DGCs such as ScrJ, ScrL, GefA, GefB, and VPA0198 (GGDEF domain-containing proteins) synthesize c-di-GMP, promoting biofilm formation and suppressing motility (23–26).

Many regulators tightly regulate c-di-GMP metabolism, while c-di-GMP reciprocally modulates the activity of certain regulators through mechanisms such as conformational changes or allosteric effects (16). This multitiered regulatory network, comprising c-di-GMP and its interacting regulators, enables bacteria to dynamically adjust their behavior and metabolism in response to environmental cues. *V. parahaemolyticus* expresses four proteins, CpsQ, CpsS, ScrO, and ScrP, whose regulatory activities are modulated by c-di-GMP (27). CpsQ, CpsS, and ScrO require c-di-GMP binding to activate transcriptional regulation, whereas ScrP retains regulatory activity even in the absence of c-di-GMP (27). CpsQ and ScrO promote biofilm formation, with ScrO strongly activating the *cpsA-J* operon (27). CpsS regulates CpsQ expression by directly controlling *cpsR* (28). Conversely, ScrP represses the O-antigen ligase gene *wzyO*, which is positively associated with biofilm formation (12, 27).

CalR, a LysR-type transcriptional regulator, regulates diverse cellular pathways in *V. parahaemolyticus*. It activates the expression of T6SS2 genes and the transcription of *cpsQ-mfpABC* and *mfpABC* (29, 30). Conversely, CalR plays a negative role in T3SS1 gene expression and TDH production (31, 32). Additionally, CalR represses swimming and swarming motility of *V. parahaemolyticus* in a calcium concentration-dependent manner (33, 34). Most importantly, both lateral flagella-mediated swarming motility and MfpABC are essential for mature biofilm formation in *V. parahaemolyticus* (5, 12). These findings suggest that CalR likely affects biofilm formation in *V. parahaemolyticus*. In this study, we demonstrate that CalR enhances biofilm formation by increasing the production of EPS, extracellular proteins, eDNA, and c-di-GMP, as well as upregulating ScrP expression.

## MATERIALS AND METHODS

### Bacterial strains and growth conditions

*V. parahaemolyticus* strain RIMD2210633 (wild type [WT]) was used in this study. A nonpolar *calR* deletion mutant (*ΔcalR*), derived from the WT strain, was constructed in a previous study (32). The recombinant plasmid pBAD33-*calR* was introduced into *ΔcalR* to yield the complementation strain *ΔcalR*/pBAD33-*calR* (C-*ΔcalR*). Control strains (WT/pBAD33 and *ΔcalR*/pBAD33) were generated by introducing pBAD33 into WT and *ΔcalR*, respectively. Single-gene deletion mutants (*Δvpa0184*, *Δvpa1429*, and *ΔscrP*) were generated by deleting the following fragments from the WT strain via homologous recombination using the suicide plasmid pDS132 (35, 36): a 156 bp fragment (nucleotides 220–395) of *vpa0184*, a 488 bp fragment (nucleotides 1444–1931) of *vpa1429*, and a 177 bp fragment (nucleotides 87–263) of *scrP*. The DNA fragment containing the coding region of *vpa1429* or *scrP*, along with a synthetic ribosome binding site (AGGAGG), was cloned into the pBAD33 vector, which contains an L-arabinose-inducible promoter and a chloramphenicol resistance gene (37). All primers used are listed in Table S1.

*V. parahaemolyticus* was grown as previously described (38, 39). Briefly, overnight cultures in 2.5% (wt/vol) heart infusion (HI) broth (BD Biosciences, USA) were diluted 50-fold into 5 mL of HI broth and incubated at 37°C with shaking (200 rpm) until reaching an optical density at 600 nm (OD_600_) of 1.4 (hereafter termed as the bacterial seed). The resulting culture was diluted 1,000-fold into 5 mL of HI broth for a third growth cycle. Bacterial cells were harvested at an OD_600_ value of 1.4. When necessary, the medium was supplemented with 50 µg/mL gentamicin, 5 µg/mL chloramphenicol, and/or 0.1% (wt/vol) L-arabinose.

### Motility assays

Swimming and swarming motility assays were performed as previously described (40, 41). Briefly, 2 µL of the bacterial seed was inoculated into a semi-solid HI plate containing 0.5% (wt/vol) Difco Noble agar (BD Biosciences) for swimming motility or spotted onto an HI plate containing 2.0% (wt/vol) Difco Noble agar for swarming motility. The diameters of the swimming and swarming areas were measured after incubation at 37°C for the indicated durations.

### Colony morphology assay

The colony morphology assay was performed as previously described (42). Briefly, an overnight bacterial culture was diluted 50-fold into 5 mL of Difco marine broth 2216 (M broth, BD Biosciences) and statically incubated at 30°C for 48 hours. After thorough mixing, 2 µL of the culture was spotted onto an HI plate or an HI plate supplemented with 5 µg/mL chloramphenicol and 0.1% (wt/vol) L-arabinose, followed by incubation at 37°C for 48 hours.

### Crystal violet staining assay

The crystal violet (CV) staining assay was performed as previously described (42). Briefly, the bacterial seed was diluted 50-fold into 2 mL of M broth supplemented with 5 µg/mL chloramphenicol and 0.1% (wt/vol) L-arabinose in a 24-well cell culture plate and incubated at 30°C with shaking (150 rpm) for 24 hours. Planktonic cells were collected to measure OD_600_ values. Surface-attached biofilm cells were washed with deionized water and stained with 0.1% CV. Bound CV was dissolved in 2.5 mL of 20% (vol/vol) acetic acid, and OD_570_ values were measured. The capacity of biofilm formation was quantified as OD_570_/OD_600_.

### Measurement of growth curves

The bacterial seed was diluted 1,000-fold into 10 mL of HI or M broth in a bacteria-free plastic centrifuge tube, mixed thoroughly, and aliquoted into a 96-well cell culture plate (200 µL per well) with 12 biological replicates per strain. Bacterial growth curves were created using a microbial growth curve analyzer (MGC-200; Ningbo Scientz Biotechnology Co. Ltd., China) by monitoring the OD_600_ values at 30 min intervals. The target temperature was 37°C for HI broth or 30°C for M broth, with an oscillation frequency of 800 rpm.

### Determination of major components of the biofilm matrix

Extracellular proteins, eDNA, and EPS in the biofilm matrix were determined as previously described (43). Briefly, the bacterial seed was diluted 50-fold into 2 mL of M broth in a 24-well cell culture plate and incubated at 30°C with shaking (150 rpm) for 24 hours. Planktonic cells were removed, and surface-attached biofilm cells were washed twice with pre-chilled phosphate buffered saline (PBS). Biofilm cells were resuspended in pre-chilled 0.01 M KCl and disrupted via sonication. After centrifugation, the supernatant was collected and filtered through a 0.22 µm pore-size membrane filter (Millipore, USA). EPS content was measured using the phenol-sulfuric acid method and expressed as OD_490_. Extracellular proteins were quantified with a Pierce BCA Protein Assay kit (Thermo Fisher Scientific, USA) according to the manufacturer’s instructions. eDNA content was determined using a M5 Pic5 Green dsDNA Assay Kit (Mei5 Biotechnology, China) and a fluorescence microplate reader (Tecan Infinite M200, Switzerland) with excitation/emission wavelengths of 485 nm/535 nm.

### Detection of Op-Tr phase variation

The Op-Tr phase variation was detected as previously described (42). Briefly, a small portion of the overnight bacterial-cell culture was streaked onto an HI plate and incubated at 37°C for 24 hours.

### RNA isolation and RNA sequencing

Bacterial cells from the third growth cycle were harvested at an OD_600_ of 1.4. Total RNA was extracted using TRIzol Reagent (Invitrogen, USA). RNA concentration and integrity were determined with a Nanodrop 2000 and agarose gel electrophoresis, respectively. rRNA removal and mRNA enrichment were performed using an Illumina/Ribo-Zero rRNA Removal Kit (bacteria) (Illumina, USA). All RNA-related manipulations, including RNA extraction, were performed in GENEWIZ Biotechnology Co. Ltd. (Suzhou, China). cDNA sequencing was performed on an Illumina HiSeq platform (8). Gene expression in *ΔcalR* (test group) was compared with that in WT (reference group). DESeq (v.1.12.4) was used to identify the differentially expressed genes (DEGs) (44), filtered with a *P* value of ≤0.01 and absolute fold change of ≥2. DEGs were further analyzed using the Gene Ontology (GO), Kyoto Encyclopedia of Genes and Genomes (KEGG) pathway, and Cluster of Orthologous Groups of Proteins (COG) database (45–47).

### Real-time quantitative PCR

Bacterial cells from the third growth cycle were harvested at an OD_600_ of 1.4. Total RNA was extracted from WT/pBAD33, *ΔcalR*/pBAD33, and C-*ΔcalR*, respectively, using TRIzol Reagent (Invitrogen). cDNA was generated from 1 µg of total RNA with a FastKing First Strand cDNA Synthesis Kit (Tiangen Biotech, China). Quantitative PCR was performed on a LightCycler 480 (Roche, Switzerland) using SYBR Green Master Mix (Tiangen Biotech) (48). The relative expression level of each target gene was determined using the 2^−ΔΔCt^ method, with the 16S rRNA gene as the internal control.

### Luminescence assay

The luminescence assay was performed as previously described (49). Briefly, the promoter-proximal DNA region of each target gene was cloned into the pBBRlux plasmid harboring a promoterless *luxCDABE* reporter operon. The recombinant pBBRlux was then transferred into WT and *ΔcalR*, respectively, to measure Lux activity in each strain. Luminescence was measured using an Infinite 200 Pro NanoQuant (Tecan, Switzerland). Relative light unit activity was calculated as light units per OD_600_.

### Two-plasmid *lacZ* fusion reporter assay

The promoter-proximal DNA region of each target gene was cloned into the pHRP309 plasmid containing a promoter-less *lacZ* reporter gene. The recombinant pHRP309 was transferred into *Escherichia coli* 100 λpir (EC100; Epicenter, USA) harboring pBAD33-*calR* or pBAD33. Transformants were cultured in Luria-Bertani broth supplemented with 0.1% L-arabinose and 20 µg/mL chloramphenicol at 37°C with shaking (200 rpm). Bacterial cells were harvested at an OD_600_ of 1.2 and lysed to measure β-galactosidase activity in cell extracts using the β-Galactosidase Enzyme Assay System (Promega, USA). Miller units representing β-galactosidase activity were calculated as previously described (42).

### Purification of 6× His-CalR and electrophoretic mobility-shift assay

Expression and purification of His-CalR were performed as previously described (29). The dialyzed protein was concentrated to approximately 0.5 mg/mL, and its purity was confirmed by sodium dodecyl sulfate-polyacrylamide gel electrophoresis. For electrophoretic mobility shift assay (EMSA) (42), the promoter-proximal DNA region of each target gene was amplified by PCR. EMSA was performed in a 10 µL reaction mixture containing 0.5 mM EDTA, 1 mM MgCl_2_, 50 mM NaCl, 0.5 mM DTT, 10 mM Tris–HCl (pH 7.5), 0.625 µg/mL salmon sperm DNA, 100 ng target DNA, and a specific amount of His-CalR. After incubation at room temperature for 20 min, binding results were analyzed on a native polyacrylamide gel using a UV transilluminator following ethidium bromide staining.

### Quantification of c-di-GMP concentration

Intracellular c-di-GMP concentration was quantified as previously described (42). Briefly, bacterial cells from 1 mL of the third cycle of culture were harvested at an OD_600_ of 1.4 and resuspended in 2 mL of ice-cold PBS. The bacterial suspension was incubated at 100°C for 5 min, sonicated for 15 min, and centrifuged at 10,000 × *g* for 5 min. Total protein and c-di-GMP levels in the supernatant were determined using a Pierce BCA Protein Assay kit (Thermo Fisher Scientific) and a c-di-GMP Enzyme-linked Immunosorbent Assay Kit (MSKBIO, China), respectively. The c-di-GMP concentration was expressed as picomoles per gram of protein.

### Statistical methods

Biofilm phenotype-associated assays, motility assays, luminescence assays, real-time quantitative PCR (RT-qPCR), and two-plasmid *lacZ* fusion reporter assays were performed in at least three independent experiments, each with three biological replicates. Results were expressed as the mean ± standard deviation. Paired Student’s *t*-tests or two-way analysis of variance with Tukey’s post hoc corrections were applied to calculate the statistical significance, with a *P* value of <0.05 considered significant. EMSAs for each target gene were performed in at least two independent experiments.

## RESULTS

### CalR affects the growth of *V. parahaemolyticus* in HI broth

The effect of CalR on the growth of *V. parahaemolyticus* was investigated by measuring the growth curves of the WT and *ΔcalR* strains in HI (37°C) broth and M broth (30°C). As shown in Fig. S1, the growth rate of the *ΔcalR* strain in HI broth at 37°C was significantly lower than that of the WT strain from the fifth hour of incubation. In contrast, no significant difference in growth rate was observed between the *ΔcalR* and WT strains in M broth 30°C at any time point. These results suggest that CalR is required for optimal growth of *V. parahaemolyticus* in HI broth at 37°C.

### CalR inhibits swimming and swarming motility in *V. parahaemolyticus*

**Fig 1:**
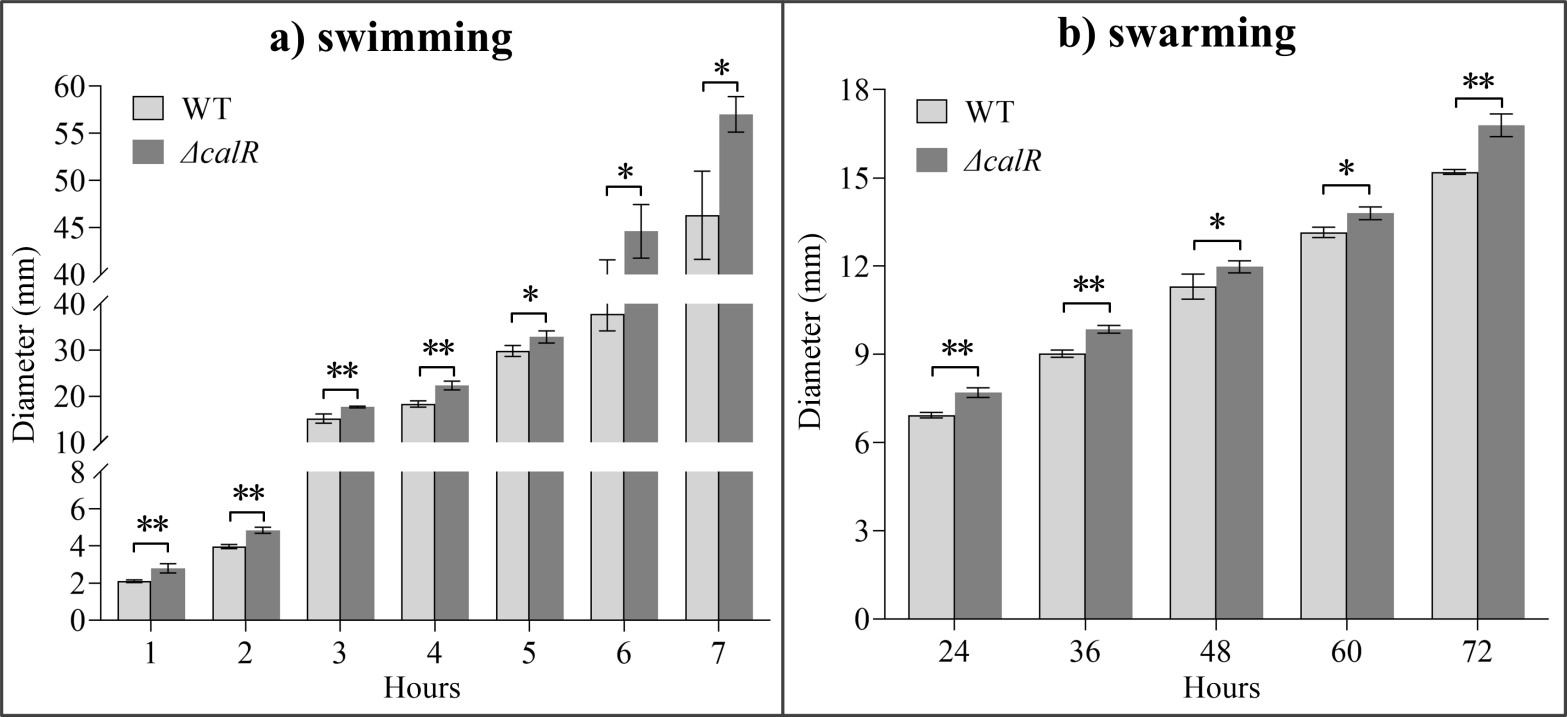
Negative regulation of CalR on swimming and swarming motility of *V. parahaemolyticus*. The swimming (**a**) or swarming (**b**) capacity of *V. parahaemolyticus* was evaluated by measuring the diameters of swimming or swarming areas. The assays were performed at least three times with five biological replicates in each time. The results are expressed as the mean ± standard deviationand analyzed by paired Student’s *t*-test. *, *P* < 0.05; **, *P* < 0.01.

CalR negatively regulates lateral flagellar gene expression and the swarming motility on the swarm plate containing 25 g HI, 15 g NaCl, and 15 g agar per liter (33). However, the expression level of CalR under low-salt conditions (0.66% NaCl) was reported to be significantly higher than under high-salt conditions (2% NaCl) (50). In this study, we investigated the effect of CalR on the swimming and swarming motility of *V. parahaemolyticus* under low-salt growth conditions (0.5% NaCl). As shown in Fig. 1, both swimming and swarming motility of *ΔcalR* were significantly enhanced compared to WT at all tested time points (*P* < 0.05), indicating that CalR also represses motility in *V. parahaemolyticus* under low-salt conditions.

### CalR promotes biofilm formation by *V. parahaemolyticus*

**Fig 2:**
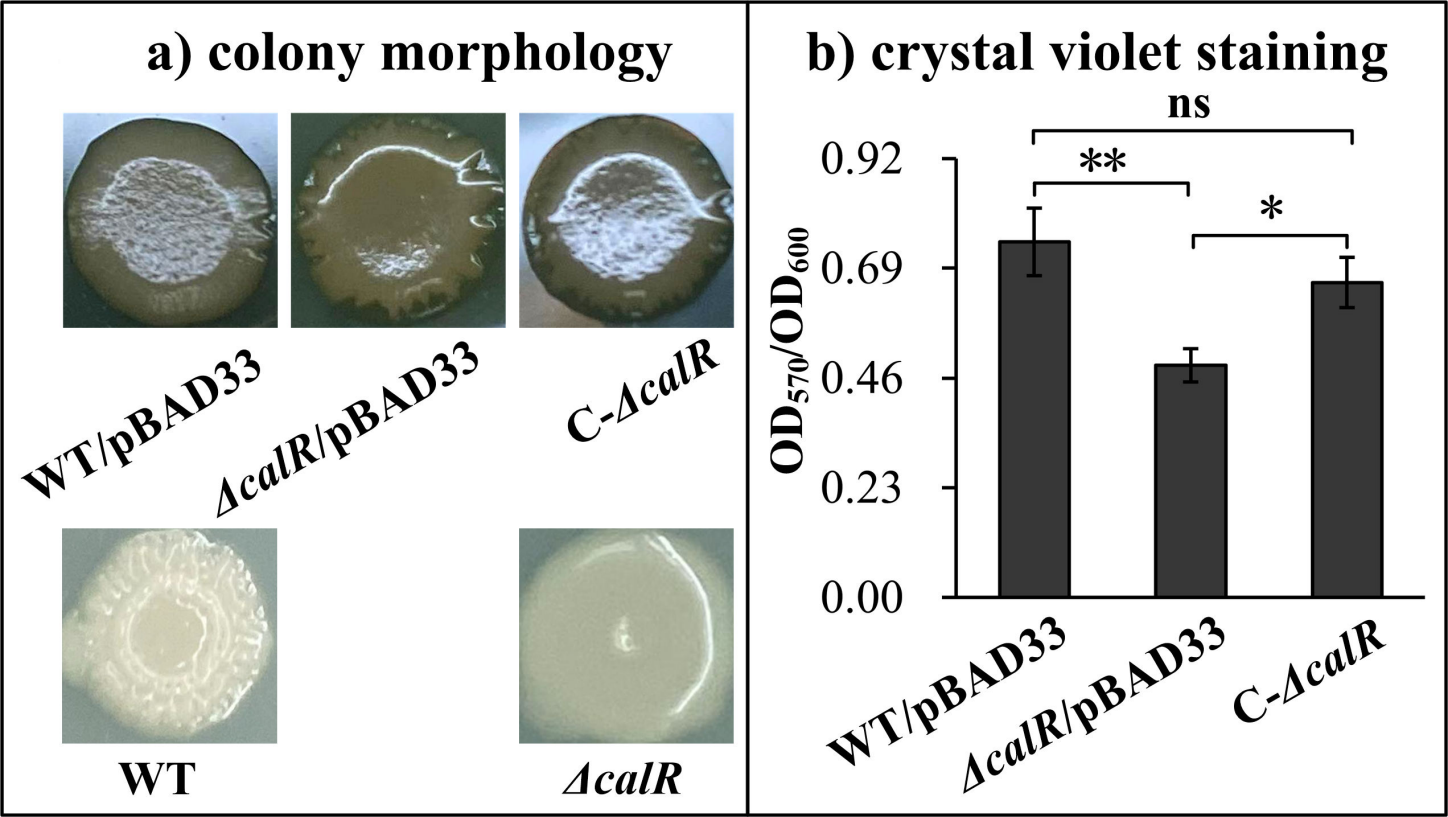
CalR promotes biofilm formation by *V. parahaemolyticus*. Biofilm formation was assessed using colony morphology (**a**) and crystal violet staining (**b**). Photographs are representative of three independent experiments with at least three replicates each. The numerical results are expressed as the mean ± standard deviation and analyzed by two-way analysis of variance. *, *P* < 0.05; **, *P* < 0.01; ns, not significant (*P* > 0.05).

Flagella-driven swimming and swarming motility are required for mature biofilm formation in *Vibrio* spp. (5, 12). Given the CalR-dependent inhibition of these motilities, we investigated whether CalR also regulates biofilm formation in *V. parahaemolyticus*. As shown in Fig. 2a, WT colonies exhibited a wrinkled morphology, whereas *ΔcalR* colonies appeared smooth. Strains carrying the complementation plasmid (C-*ΔcalR*) or the empty vector (WT/pBAD33) showed slight wrinkling–much less pronounced than WT but more than *ΔcalR*/pBAD33. Notably, the colony morphologies of C-*ΔcalR* and WT/pBAD33 were nearly identical, suggesting that chloramphenicol (plasmid selection) and L-arabinose (inducer) partially attenuated EPS production. Consistent with this, *ΔcalR*/pBAD33 displayed significantly reduced CV staining compared to WT/pBAD33 and C-*ΔcalR* (*P* < 0.05), while C-Δ*calR* restored CV staining to levels resembling WT/pBAD33 (Fig. 2b). Together, these results indicate that CalR positively regulates biofilm formation by *V. parahaemolyticus*.

### Deletion of *calR* reduces the contents of the biofilm matrix

**Fig 3:**
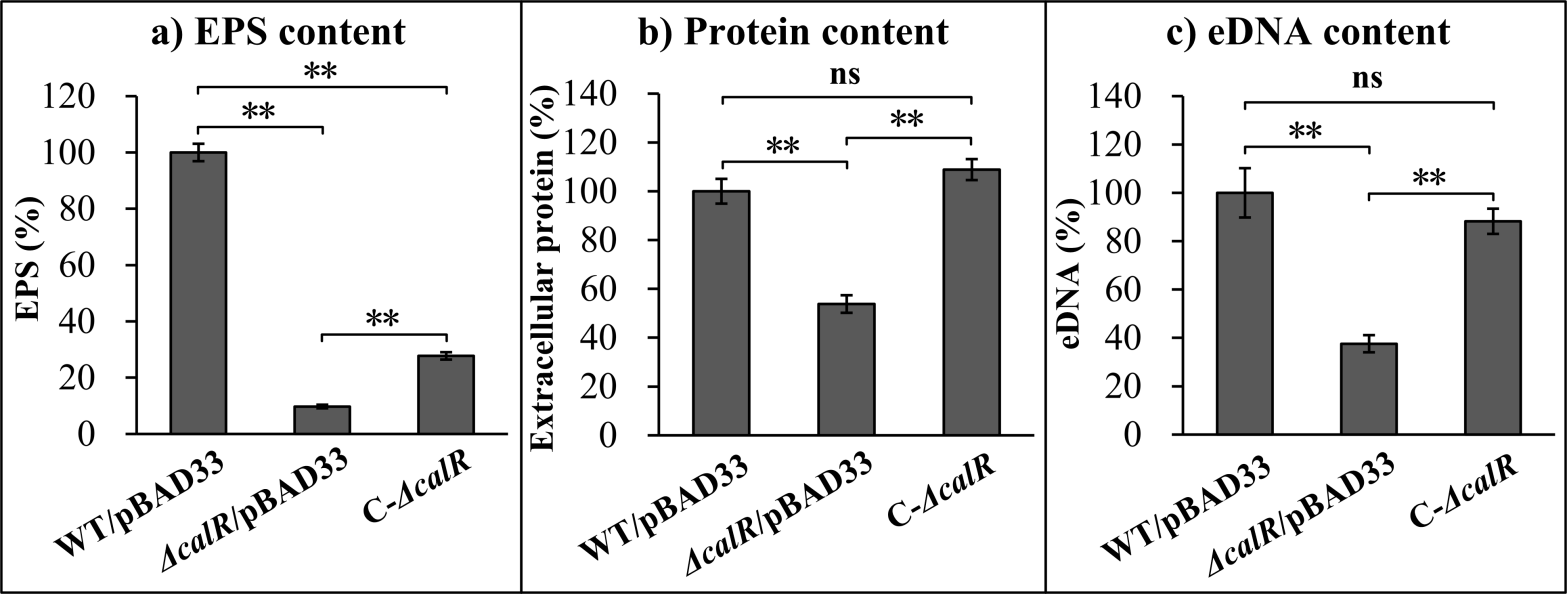
CalR promotes the production of biofilm matrix component. The effect of CalR on the main component of biofilm matrix was measured by detecting the relative contents of EPS (**a**), extracellular proteins (**b**), and eDNA (**c**). **, *P* < 0.01; ns, *P* > 0.05.

The biofilm matrix, which plays a key role in the stability of the biofilm, is mainly composed of EPS, extracellular proteins, eDNA, lipids, and water (6). In the biofilm formed by *ΔcalR*/pBAD33, the contents of EPS, extracellular proteins, and eDNA are all significantly lower than those in the biofilms formed by WT/pBAD33 and C-*ΔcalR* (Fig. 3). In the biofilm formed by C-*ΔcalR*, the contents of extracellular proteins and eDNA are restored to levels comparable to those in WT/pBAD33. However, although the content of EPS in the C-*ΔcalR* biofilm increases, it remains significantly lower than that in WT/pBAD33, only partially recovering the phenotype. Therefore, deletion of *calR* reduces the content of the biofilm matrix.

### CalR has no regulatory effect on Op-Tr phase variation

The Op-Tr phase variation is directly associated with CPS production levels, where elevated CPS production results in Op colony morphology (51). Since CPS restricts biofilm formation by limiting biofilm size (52), we investigated whether CalR regulates CPS production in *V. parahaemolyticus*. As shown in Fig. S2, both the WT and *ΔcalR* strains formed Op lawns, whereas the *ΔopaR* control strain (42) exhibited Tr lawns. These findings indicate that CalR does not influence Op-Tr phase variation or CPS production in *V. parahaemolyticus*.

### Screening biofilm formation-associated genes regulated by CalR using RNA sequencing

**Fig 4:**
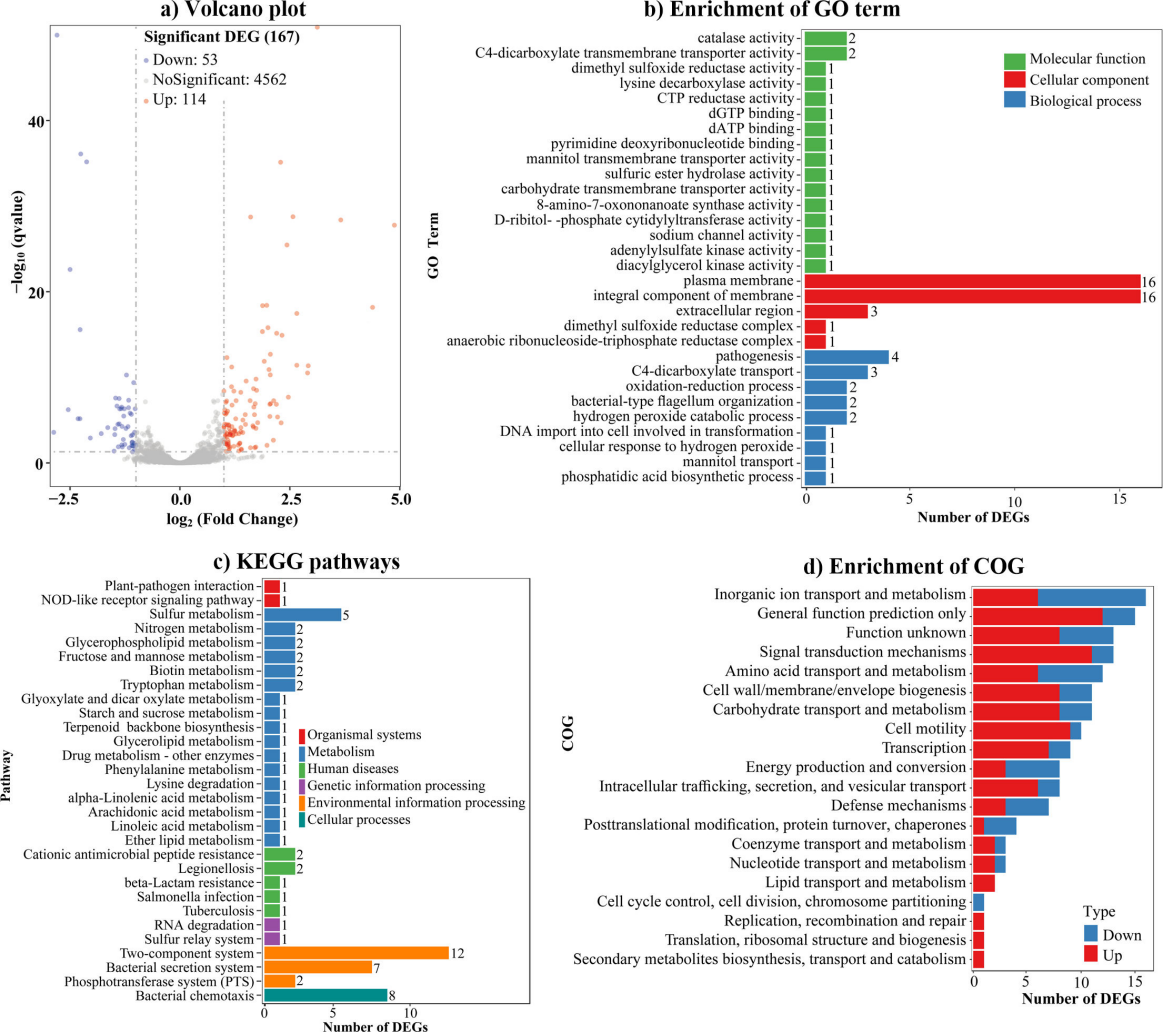
CalR regulates global gene expression. (**a**) Volcano plot. Red, green, and black points represent the upregulated, downregulated, and no-differential expressed genes, respectively. (**b**) Enrichment of Gene Ontology (GO) term. (**c**) Enrichment of Kyoto Encyclopedia of Genes and Genomes (KEGG). (**d**) Enrichment of Cluster of Orthologous Groups of Proteins (COG). The number on the top of each bar in panels b and c indicates the number of DEGs.

To elucidate the regulatory mechanism of CalR on *V. parahaemolyticus* biofilm formation, the transcriptional profiles of *ΔcalR* (test) and WT (reference) were compared using RNA sequencing (RNA-seq). As shown in Fig. 4a and Table S2, 167 genes were differentially regulated by CalR under the tested growth conditions. Of these, 53 genes were positively regulated, and 114 were negatively regulated by CalR. The enrichment of GO terms revealed that DEGs were associated with three categories: molecular function (16 GO terms and 18 DEGs), cellular component (5 GO terms and 37 DEGs), and biological process (9 GO terms and 17 DEGs) (Fig. 4b). The most significant enrichment occurred in cellular components, particularly the plasma membrane (16 DEGs) and integral membrane components (16 DEGs). The KEGG enrichment results indicated that 66 DEGs were involved in organismal systems, metabolism, human diseases, genetic information processing, environmental information processing, and cellular processes (Fig. 4c). The most represented pathways included the two-component system (12 DEGs), bacterial chemotaxis (8 DEGs), bacterial secretion system (7 DEGs), and sulfur metabolism (5 DEGs). COG enrichment analysis classified DEGs into 20 functional categories, with the most enrichment in inorganic ion transport and metabolism (Fig. 4d). These results demonstrate that CalR regulates global gene expression in *V. parahaemolyticus*.

Among the 167 DEGs, the genes of particular interest include *scrP*, *vpa0184*, *vpa0198*, *vpa1429*, *vp2258*, and *vp2811*, as these are either known or strongly predicted to be associated with biofilm formation (Table 1). Of these, *scrP* encodes a c-di-GMP-binding regulator that represses *wzyO* expression, a gene encoding an O-antigen ligase critical for biofilm formation in *V. parahaemolyticus* BB22 (12, 27); *vpa0184*, *vpa0198*, and *vpa1429* encode putative c-di-GMP-metabolizing enzymes; and *vpa0198* has been experimentally confirmed to modulate c-di-GMP levels and biofilm formation in *V. parahaemolyticus* (25); *vp2258* and *vp2811* are polar flagellar genes. Notably, RNA-seq data showed that genes within the *cpsA-J* and *scvA-O* operons were not differentially expressed in the *ΔcalR* mutant; however, the mutant displayed a smooth colony phenotype (Fig. 2a), which is directly linked to reduced EPS production. Since wrinkled/smooth colony morphology is directly associated with EPS production—and *cpsA-J* and *scvA-O* are established EPS biosynthesis-related loci in *V. parahaemolyticus* (15)—the smooth phenotype of *ΔcalR* strongly suggests that CalR positively regulates EPS production. This regulation likely occurs potentially through transcriptional control of *cpsA-K* and *scvA-O*. Therefore, *scrP*, *vpa0184*, *vpa0198*, *vpa1429*, *cpsA*, and *scvE* were selected as target genes for further experimental validation.

**TABLE 1:** Selected DEGs

Locus tag	Old locus tag	Gene name	Fold change	Product
Putative regulators				
VP_RS01775	VP0368		2.6090	MltR family transcriptional regulator
VP_RS05325	VP1093		2.2885	MarR family transcriptional regulator
VP_RS13295	VP2710	*scrP*	3.1840	Helix-turn-helix transcriptional regulator
c-di-GMP metabolism				
VP_RS16165	VPA0184		3.3182	GGDEF domain-containing protein
VP_RS16225	VPA0198		2.2303	GGDEF domain-containing protein
VP_RS22000	VPA1429		0.3203	EAL domain-containing protein
Antioxidative enzymes				
VP_RS18855	VPA0768	*katG1*	0.3965	Catalase/peroxidase HPI
VP_RS21945	VPA1418	*katE1*	0.3596	Catalase
T3SS1				
VP_RS07975	VP1656	*vopD*	2.1965	Type III secretion system translocon subunit VopD
VP_RS07980	VP1657	*vopB*	2.1938	Type III secretion system translocon subunit VopB
VP_RS07985	VP1658	*vcrH*	2.0010	SycD/LcrH family type III secretion system chaperone VcrH
VP_RS07995	VP1660	*vcrG*	2.0097	LcrG family type III secretion system chaperone VcrG
VP_RS08000	VP1661	*vcrR*	2.0918	LcrR family type III secretion system chaperone VcrR
VP_RS08010	VP1663	*vscY*	2.2727	Type III secretion system chaperone VscY
VP_RS08015	VP1664	*vscX*	2.0965	Type III secretion system protein VscX
VP_RS08040	VP1669	*vscO*	2.0284	Type III secretion system central stalk protein VscO
VP_RS08045	VP1670	*vscP*	2.0005	Type III secretion system needle length determinant VscP
VP_RS08050	VP1671	*vscQ*	2.1180	SctQ family type III secretion system cytoplasmic ring protein VscQ
VP_RS08055	VP1672	*vscR*	2.0377	SctR family type III secretion system export apparatus subunit VscR
VP_RS08065	VP1674	*vscT*	2.5337	SctT family type III secretion system export apparatus subunit VscT
VP_RS08070	VP1675	*vscU*	2.1462	SctU family type III secretion system export apparatus subunit VscU
VP_RS08095	VP1680	*vopQ*	2.1987	Type III secretion system effector VopQ
VP_RS08100	VP1682	*vecA*	2.6092	CesT family type III secretion system chaperone VecA
VP_RS08105	VP1683	*vopR*	2.1611	Type III secretion system effector VopR
VP_RS08110	VP1684		2.1935	Type III secretion system chaperone
VP_RS08120	VP1687		2.0119	CesT family type III secretion system chaperone
VP_RS08130	VP1689	*vscK*	2.1728	SctK family type III secretion system sorting platform protein VscK
VP_RS08160	--		2.0421	EscE/YscE/SsaE family type III secretion system needle protein co-chaperone
VP_RS08185	VP1699	*exsA*	2.4099	Type III secretion system transcriptional regulator ExsA
Polar flagellum				
VP_RS10965	VP2258		2.6137	Flagellin
VP_RS13775	VP2811		2.1469	sel1 repeat family protein
Outer membrane proteins				
VP_RS04920	VP1008		2.5070	Porin
VP_RS07865	VP1634		2.0509	TolC family outer membrane protein

### CalR regulates *scrP*, *vpa0184*, *vpa0198*, *vpa1429*, *cpsA*, and *scvE* transcription

**Fig 5:**
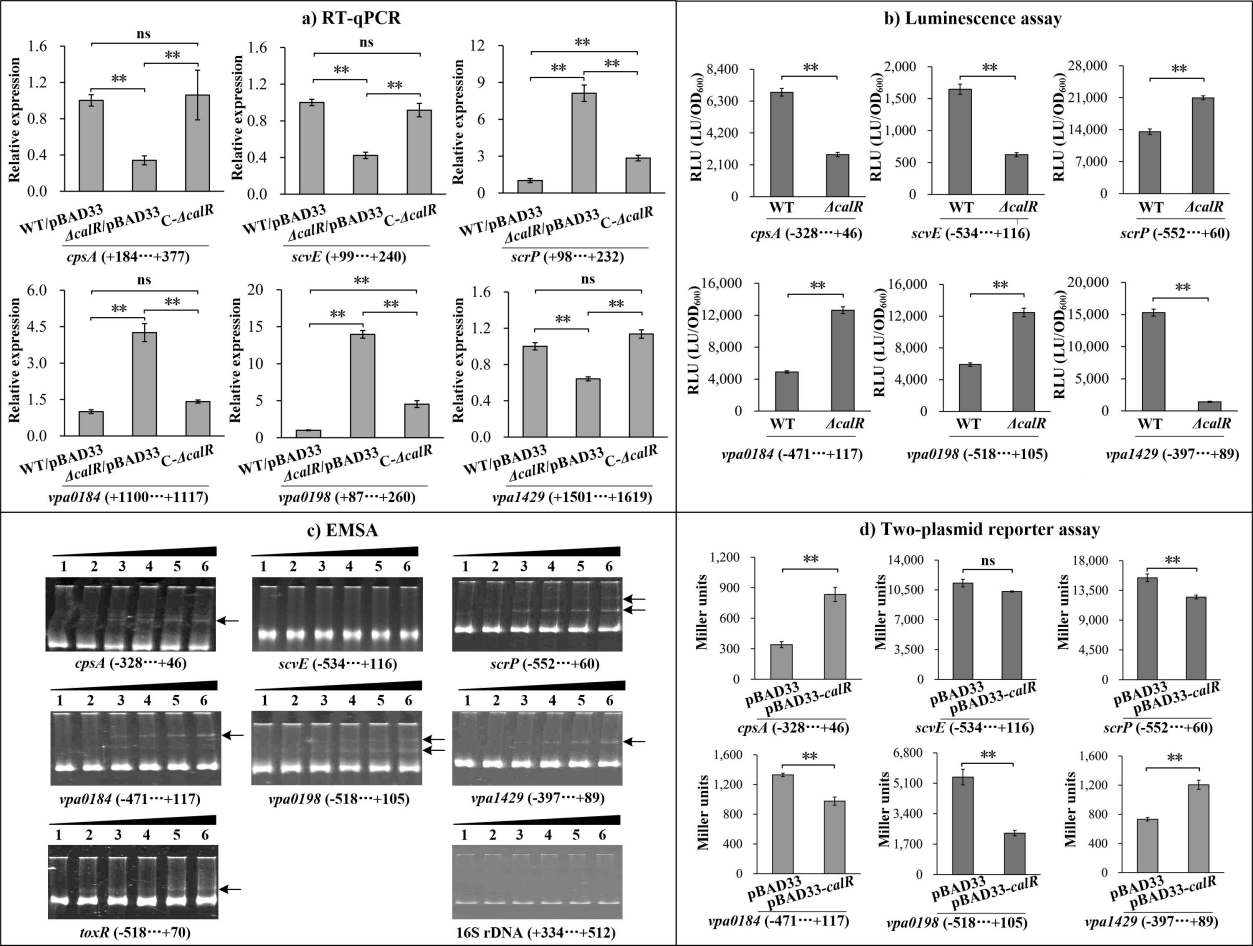
Regulation of *scrP*, *vpa0184*, *vpa0198*, *vpa1429*, *cpsA*, and *scvE* by CalR. *V. parahaemolyticus* was grown in HI broth, and bacterial cells were harvested at an OD_600_ value of 1.4. Negative and positive numbers in brackets indicate the nucleotide positions upstream and downstream of each target gene, respectively. **, *P* < 0.01; ns, *P* > 0.05. (**a**) Quantitative PCR. The relative mRNA levels of each target gene were examined and compared among WT/pBAD33, *ΔcalR*/pBAD33, and C-*ΔcalR*. (**b**) Luminescence assay. The regulatory DNA region of each target gene was cloned into the pBBRlux plasmid and then transferred into *ΔcalR* and WT, respectively, to determine the luminescence activities in the cellular extracts. The luminescence activity (relative light unit [RLU]) was calculated as light units/OD_600_. (**c**) EMSA. The promoter proximal DNA region of each target gene was incubated with increasing amounts of purified His-CalR and then subjected to 6% (wt/vol) polyacrylamide gel electrophoresis. DNA bands were visualized using ethidium bromide staining. Lanes 1–6 contain 0, 2.78, 5.56, 8.33, 11.10, and 13.80 pmol of His-CalR, respectively. The arrows indicate the shift bands. (**d**) Two-plasmid *lacZ* reporter assay. pBAD33-*calR* (or pBAD33) and a recombinant *lacZ* plasmid were simultaneously introduced into *E. coli* 100 λpir (Epicenter). The promoter activities (Miller units) of each target gene in the cellular extracts were determined using a β-Galactosidase Enzyme Assay System (Promega, USA) according to the manufacturer’s instructions.

The results of RT-qPCR showed that the mRNA levels of *cpsA*, *scvE*, and *vpa1429* were significantly decreased in *ΔcalR*/pBAD33 compared to WT/pBAD33, whereas those of *scrP*, *vpa0184*, and *vpa0198* were markedly increased (Fig. 5a). In contrast, C-*ΔcalR* exhibited restored expression levels for all these genes (Fig. 5a). As further determined by luminescence assay (Fig. 5b), the promoter activities of *cpsA*, *scvE*, and *vpa1429* were significantly decreased in the *ΔcalR* strain compared to the WT strain (*P* < 0.01), whereas those of *vpa0184* and *vpa0198* were remarkably increased. In contrast, the promoter activity of *scrP* in the *ΔcalR* strain showed only a statistically significant but sub-twofold change in expression relative to the WT strain. The results of EMSA showed that His-CalR was able to dose-dependently bind the upstream DNA fragments of *cpsA*, *scrP*, *vpa0184*, *vpa0198*, and *vpa1429*, as well as *toxR* (positive control [31]) but did not bind the promoter DNA region of *scvE* or the coding region of 16S rRNA (negative control) (Fig. 5c). In addition, the results of the two-plasmid *lacZ* reporter assay demonstrated that expression of *calR* from pBAD33-*calR* in EC100 significantly enhanced the promoter activities of *cpsA* and *vpa1429* but remarkably decreased those of *scrP*, *vpa0184*, and *vpa0198*, while it had no effect on that of *scvE* (Fig. 5d). These results suggest that CalR directly regulates the promoter activities of *cpsA*, *scrP*, v*pa0184*, *vpa0198*, and *vpa1429* but indirectly controls that of *scvE*. Taken together, CalR directly activates the transcription of *cpsA* and *vpa1429* but indirectly activates *scvE* transcription; CalR represses the transcription of *scrP*, *vpa0184*, and *vpa0198* in a direct manner.

### CalR, VPA0184, and VPA1429 contribute to the c-di-GMP metabolism of *V. parahaemolyticus*

**Fig 6:**
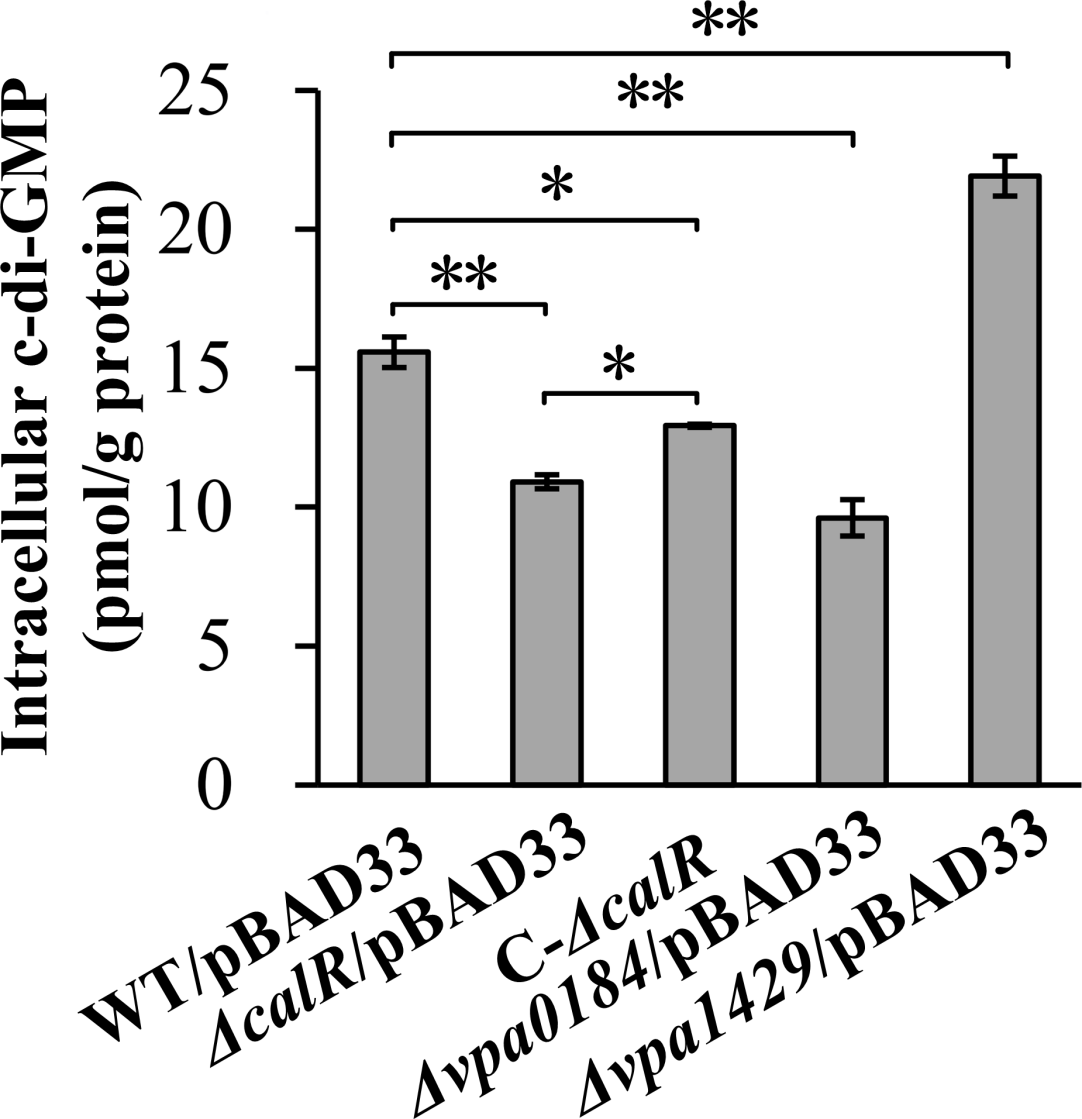
Regulation of c-di-GMP by CalR, VPA0184, and VPA1429. *V. parahaemolyticus* strains were grown in HI broth, and bacterial cells were harvested at an OD_600_ value of 1.4. Intracellular c-di-GMP levels were determined using a c-di-GMP enzyme-linked immunosorbent assay kit. The data were expressed as the mean ± standard deviation of at least three independent experiments with four biological replicates in each time. The results were analyzed by two-way analysis of variance. **, *P* < 0.01; *, *P* < 0.05.

VPA0184 and VPA0198 are proteins containing GGDEF domains, while VPA1429 is a protein with an EAL domain. Previously, we demonstrated that VPA0198 is involved in c-di-GMP metabolism and biofilm formation in *V. parahaemolyticus* (25). However, the roles of VPA0184 and VPA1429 in c-di-GMP metabolism had not been investigated prior to this study. In this study, the GGDEF domain and EAL domain were deleted from VPA0184 and VPA1429, respectively. Thereafter, their roles in c-di-GMP metabolism were assessed by the measurement of intracellular c-di-GMP levels in *Δvpa0184*/pBAD33, *Δvpa1429*/pBAD33, and WT/pBAD33. As shown in Fig. 6, intracellular c-di-GMP levels in *Δvpa0184*/pBAD33 were significantly reduced, whereas levels in *Δvpa1429*/pBAD33 were significantly enhanced compared to WT/pBAD33 (*P* < 0.01). These results suggest that VPA0184 synthesizes c-di-GMP, while VPA1429 degrades it. Additionally, the c-di-GMP levels in *ΔcalR*/pBAD33 were significantly decreased compared to WT/pBAD33, whereas C-*ΔcalR* exhibited restored c-di-GMP levels (Fig. 6). These results indicate that CalR plays a positive regulatory role in c-di-GMP metabolism in *V. parahaemolyticus*.

### VPA1429 and ScrP, but not VPA0184, regulate biofilm formation by *V. parahaemolyticus*

**Fig 7:**
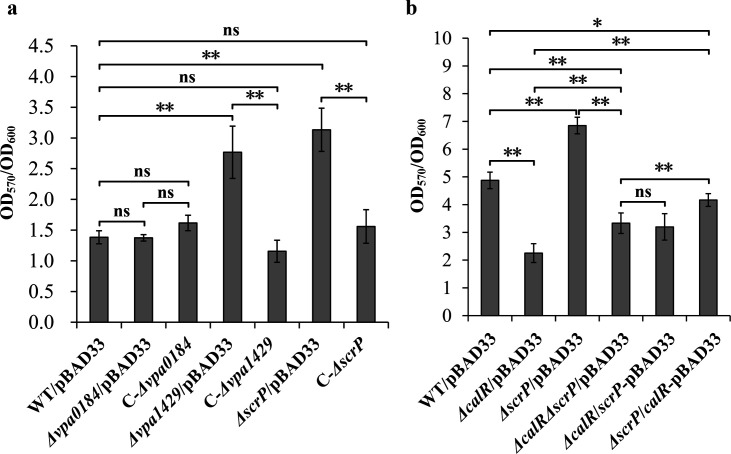
VPA1429 and ScrP, but not VPA0184, have regulatory roles in biofilm formation by *V. parahaemolyticus*. The roles of VPA0184, VPA1429, and ScrP in biofilm formation were assessed using crystal violet staining assays (**a and b**). The results are expressed as the means ± standard deviation of at least three independent experiments with five biological replicates in each time. Two-way analysis of variance with Tukey’s post hoc corrections was employed to calculate the statistical significance. ns, *P* > 0.05; *, *P* < 0.05; **, *P* < 0.01.

In this study, the roles of VPA0184, VPA1429, and ScrP in biofilm formation were assessed using CV staining assays. As shown in Fig. 7a, the CV staining results of *Δvpa0184*/pBAD33 showed no significant difference compared to those of WT/pBAD33 and C-*Δvpa0184*, indicating that VPA0184 does not affect biofilm formation. In contrast, the CV staining results of *Δvpa1429*/pBAD33 and *ΔscrP*/pBAD33 were significantly higher than those of WT/pBAD33, while the results for C-*Δvpa1429* and C-*ΔscrP* showed no significant difference compared to WT/pBAD33. These suggest that VPA1427 and ScrP inhibit biofilm formation by *V. parahaemolyticus*. To investigate how CalR regulates biofilm formation via ScrP, we constructed a double-gene mutant strain (*ΔcalRΔscrP*) and strains overexpressing CalR in *ΔscrP* or ScrP in *ΔcalR*. As shown in Fig. 7b, CV staining assays revealed that *ΔcalR*/pBAD33 exhibited significantly lower biofilm formation than WT/pBAD33, whereas *ΔscrP*/pBAD33 showed significantly higher biofilm formation (Fig. 7b), consistent with the above findings. The *ΔcalRΔscrP*/pBAD33 strain displayed biofilm levels significantly lower than *ΔscrP*/pBAD33 and WT/pBAD33 but higher than Δ*calR*/pBAD33, indicating that while CalR absence reduces biofilm formation, simultaneous ScrP deletion partially restores biofilm formation, though not to WT levels. Overexpression of *scrP* in *ΔcalR* resulted in biofilm levels similar to *ΔcalRΔscrP*/pBAD33, suggesting ScrP’s inhibitory effect requires CalR. Conversely, overexpressing *calR* in *ΔscrP* significantly enhanced biofilm formation compared to *ΔcalR*/pBAD33 and *ΔcalRΔscrP*/pBAD33, though it remained weaker than WT/pBAD33. These results suggest that the absence of ScrP had already elevated the biofilm-forming ability to a higher level, and overexpression of CalR, although further promoting biofilm formation, might be limited by other unknown factors. Taken together, CalR and ScrP exhibit a complex regulatory interplay in biofilm formation. ScrP acts as a negative regulator, while CalR positively regulates biofilm formation by suppressing ScrP expression. Their interaction critically modulates *V. parahaemolyticus* biofilm-forming capacity.

## DISCUSSION

Previously, CalR was demonstrated to inhibit both swimming and swarming motility, as well as polar and lateral flagellar gene expression, in response to calcium concentrations under growth conditions containing 2% (wt/vol) NaCl (33, 34). In this study, CalR was also shown to repress the swimming and swarming motility of *V. parahaemolyticus* under low-salt growth conditions (Fig. 1). However, CalR was shown to regulate the transcription of only two polar flagellar genes (Table 1), and the growth rate of *ΔcalR* was significantly lower than that of WT under low-salt growth conditions (Fig. S1). Therefore, it is difficult to explain the mechanisms by which CalR inhibits the swimming and swarming motility of *V. parahaemolyticus*. We previously showed that ToxR directly activates *calR* transcription, while CalR, in turn, directly inhibits *toxR* transcription (31). ToxR promotes both swimming and swarming motility in *V. parahaemolyticus* (53). Therefore, further research is needed to determine whether CalR-dependent motility repression is mediated by ToxR. Notably, CalR-dependent inhibition of swarming motility is modest, as confirmed in this study (Fig. 1). A previous study showed that CalR expression is induced under low-salt conditions (50). However, the effect observed here is not more severe than that shown in previous work (33). Gode-Potratz and colleagues used the BB22 strain (33), while the strain used in this study is RIMD2210633. Although both strains are positive for TDH, there are certain differences in their genomes (54). BB22 contains approximately 300 unique genes, whereas RIMD2210633 has around 400 unique genes (54). There are also differences in gene function between these two strains. For example, in BB22, OpaR positively regulates c-di-GMP metabolism and biofilm formation, while in RIMD2210633, OpaR acts as a negative regulator (55, 56). Therefore, the most likely cause of these differences is the use of different strains in the two studies.

Flagella-mediated motilities are required for the mature biofilm formation of *V. parahaemolyticus* (5, 12). Therefore, CalR-dependent motility inhibition suggested that CalR might regulate biofilm formation by *V. parahaemolyticus*. Indeed, the data showed that CalR positively regulated both the production of EPS and biofilm formation by *V. parahaemolyticus* (Fig. 2). The *cpsA-K* and *scvA-O* gene clusters are responsible for EPS production in *V. parahaemolyticus* (7). Our findings demonstrate that CalR directly activates *cpsA* transcription and indirectly activates *scvE* transcription (Fig. 6). These results may explain why the *calR* mutant displayed smooth colonies on agar plates (Fig. 2a). Additionally, biofilms of *ΔcalR* contained less extracellular proteins and eDNA than those of WT (Fig. 3). As extracellular proteins and eDNA are key components of the biofilm matrix (6), these indicate that CalR promotes biofilm formation in *V. parahaemolyticus* by enhancing the production of EPS, extracellular proteins, and eDNA within the matrix. However, it remains unclear whether the reduced matrix content in *ΔcalR* arises primarily from impaired matrix synthesis or from structural biofilm defects that compromise matrix retention. We propose that both mechanisms may contribute. First, CalR absence directly reduces matrix component synthesis, destabilizing biofilm structure. This is supported by CalR’s direct positive regulation of *cpsA-J* transcription (Fig. 5), suggesting that *calR* deletion directly reduces EPS production. Second, structural biofilm defects may reduce matrix retention, leading to increased release of components into the culture medium. However, this latter hypothesis requires further validation.

Previously, numerous regulators have been shown to regulate biofilm formation in *V. parahaemolyticus* by controlling the transcription of *cpsA-K* and/or *scvA-O* gene clusters. For example, CpsS is a repressor of *cpsR*; CpsR is an activator of *cpsQ*; and CpsQ is a direct activator of *cpsA* and biofilm formation (14, 28). Similarly, ScrO induces the promoter activity of *cpsA* and biofilm formation (27). AphA, which is the master quorum sensing (QS) regulator operating at low cell density, directly activates the transcription of *scvE* and biofilm formation, whereas OpaR, which is the master QS regulator operating at high cell density, directly represses the transcription of *cpsA* and *scvE*, thereby inhibiting biofilm formation (42, 57). Therefore, the findings presented in this study expand the known regulatory network governing EPS production and biofilm formation in *V. parahaemolyticus*.

RNA-seq analysis identified 167 genes regulated by CalR (Fig. 4a; Table S2). Of these, *scrP* was repressed directly by CalR (Fig. 5). ScrP, a c-di-GMP binding regulator, exhibited minimal impact on *cpsA* expression and biofilm formation in *V. parahaemolyticus* BB22 but repressed *wzyO* expression, a gene implicated in biofilm formation (12, 27). Although the mechanistic details remain unclear, CV staining assays demonstrated that ScrP negatively regulates biofilm formation by *V. parahaemolyticus* RIMD2210633 (Fig. 7). Previous studies have noted discrepancies between BB22 and RIMD2210633, which may stem from their genomic differences (42, 55). CalR also regulated the transcription of *vpa0184*, *vpa0198*, and *vpa1429*, all of which are involved in c-di-GMP metabolism (Fig. 6 [25]), thereby promoting c-di-GMP production. However, VPA0184, a GGDEF-domain containing protein predicted to function as a DGC, showed no detectable effect on biofilm formation (Fig. 7), despite its confirmed role in c-di-GMP synthesis (Fig. 6). This apparent contradiction could arise from differences in cultivation conditions between c-di-GMP measurement and CV staining assays. Additionally, VPA0184 might not serve as the primary DGC under the experimental growth conditions, given the functional redundancy of numerous DGCs in *V. parahaemolyticus* (17). For instance, overexpression of certain c-di-GMP-metabolizing enzymes, such as VCA0560 in *V. cholerae* (58), is required to observe phenotypic effects. Thus, further investigation is needed to determine whether overexpressed VPA0184 influences biofilm formation. In contrast to VPA0184, VPA1429 is a putative PDE that degrades c-di-GMP. Deletion of *vpa1429* increased intracellular c-di-GMP levels and enhanced biofilm formation (Fig. 6 and 7). Conversely, VPA0198 is a DGC that synthesizes c-di-GMP and promotes biofilm formation (25). Intriguingly, while CalR promotes c-di-GMP metabolism—with its mutation lowering c-di-GMP levels and weakening biofilm formation—it paradoxically upregulates VPA1429 (a PDE) and downregulates VPA0184 and VPA0198 (DGCs). These seemingly conflicting observations suggest that CalR’s impact on c-di-GMP homeostasis extends beyond these three genes. CalR likely regulates a broader network of c-di-GMP-related genes, and the c-di-GMP pool reflects the combined expression changes across this network. For example, even though transcription of *vpa0184*, *vpa0198*, and *vpa1429* is altered in a *calR* mutant, the overall reduction in c-di-GMP levels may result from compensatory regulation of other DGCs or PDEs. CalR appears to modulate the c-di-GMP metabolic pool by orchestrating the transcription of multiple DGC and PDE genes. The high functional redundancy of c-di-GMP-metabolizing enzymes in *V. parahaemolyticus* likely underpins the complexity of these regulatory outcomes (17).

Transcriptome data revealed that two antioxidative genes, *katG1* and *katE1*, were shown to be positively regulated by CalR (Table 1), a relationship further confirmed by RT-qPCR (data not shown). KatE1 is the main catalase for detoxifying extrinsic hydrogen peroxide during the log-phase growth, whereas KatG1 plays an antioxidative role in the stationary phase and under starved grown conditions (59, 60). Therefore, CalR could be required for oxidative stress resistance in *V. parahaemolyticus*. Additionally, *vp1008*, which encodes a putative porin protein, and *vp1634*, which encodes a TolC family outer membrane protein, were negatively regulated by CalR (Table 1), as validated by RT-qPCR (data not shown). TolC-like proteins play important roles in virulence and multidrug resistance in pathogenic bacteria (61). Porin proteins are also required for the antimicrobial resistance (62). This implies that CalR may also modulate antimicrobial resistance in *V. parahaemolyticus*. However, the mechanisms underlying CalR’s regulation of these genes remain unknown.

**Fig 8:**
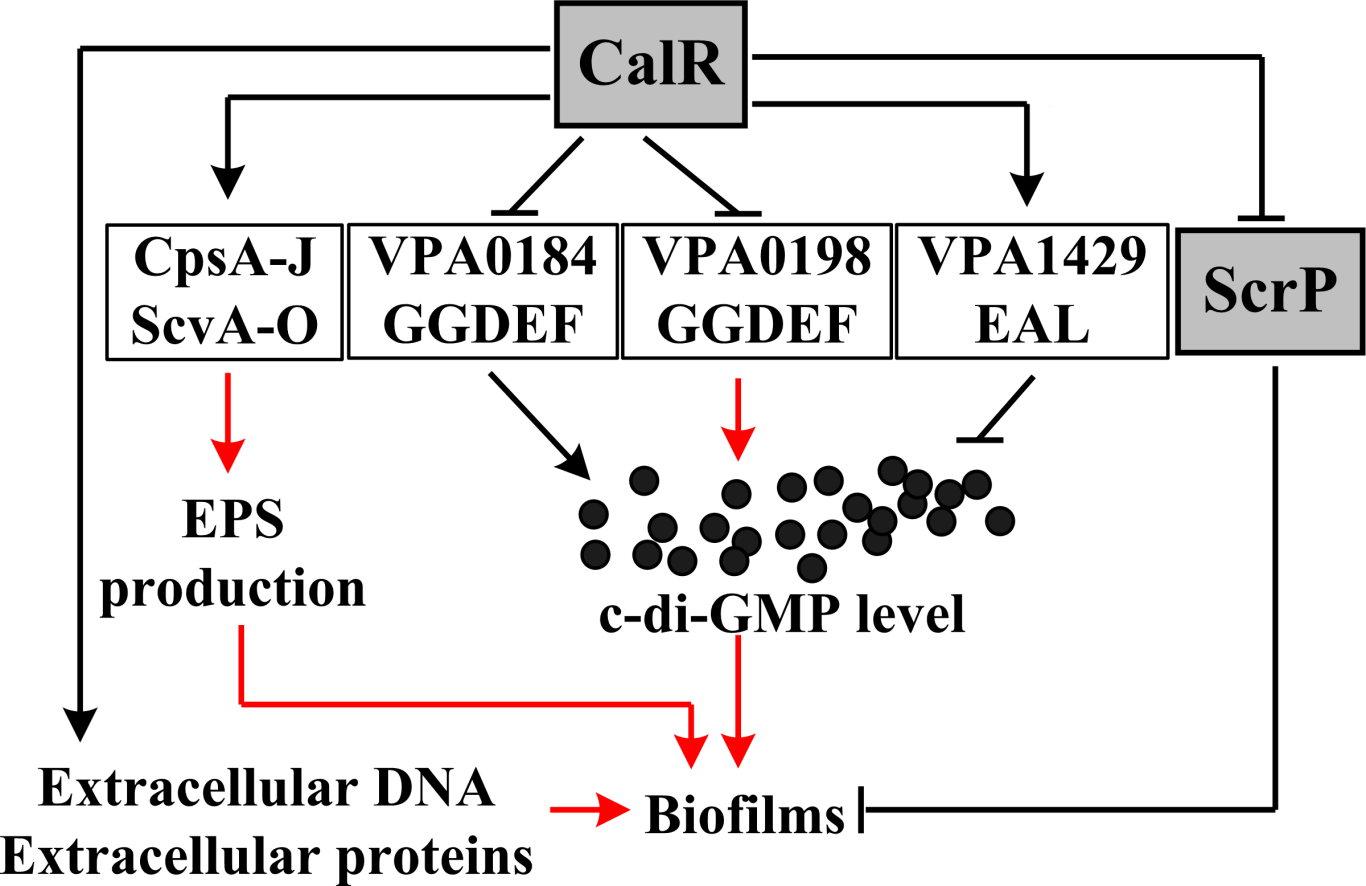
A regulatory circuit associated with CalR-dependent biofilm formation. The arrows indicate positive regulation, whereas the T-junctions represent negative regulation. The black dots indicated c-di-GMP. The regulatory relationships represented by black lines are demonstrated in this work, while those indicated by red lines are supported by the previous studies (5, 7, 9, 25).

In conclusion, our findings demonstrate that CalR positively regulates the production of EPS, extracellular proteins, and eDNA, thereby promoting biofilm formation in *V. parahaemolyticus*. However, CalR does not affect the CPS production in this organism. Transcriptomic analysis revealed that CalR regulates 167 genes, including *scrP*, *vpa0184*, *vpa0198*, and *vpa1429*. Among these, ScrP represses biofilm formation, while *vpa0184*, *vpa0198*, and *vpa1429* are potentially implicated in c-di-GMP metabolism. CalR directly represses the transcription of *scrP*, *vpa0184*, and *vpa0198* but activates *vpa1429* transcription in a direct manner, thereby enhancing the production of c-di-GMP. In addition, CalR activates the transcription of *katG1* and *katE1*, which are required for oxidative stress resistance, but represses the transcription of *vp1008* and *vp1634*, which encode putative outer membrane proteins that may contribute to antimicrobial resistance. These results highlight the need to further investigate CalR’s regulatory mechanisms in oxidative stress resistance and drug resistance. Collectively, our findings demonstrate that CalR positively regulates biofilm formation in *V. parahaemolyticus* through multiple ways (Fig. 8): (i) enhancing the production of EPS, extracellular proteins, and eDNA; (ii) stimulating the biosynthesis of c-di-GMP via transcriptional regulation of *vpa0184*, *vpa0198*, and *vpa1429*; and (iii) suppressing the transcription of *scrP*. These findings expand the known regulatory network governing biofilm formation in *V. parahaemolyticus*.

## Data Availability

The original data presented in the study are included in the article and its supplemental material. The raw data of RNA sequencing have been deposited in the National Center for Biotechnology Information repository under accession number PRJNA1057610.

## SUPPLEMENTAL MATERIAL

Supplemental figures aem.00724-25-s0001.docx Fig. S1, growth curves of WT and ΔcalR strains; Fig. S2, CalR has no regulatory effect on Op-Tr phase variation.

Table S1 aem.00724-25-s0002.docx Oligonucleotide primers used in this study.

Table S2 aem.00724-25-s0003.xlsx CalR regulon.

ASM does not own the copyrights to Supplemental Material that may be linked to, or accessed through, an article. The authors have granted ASM a non-exclusive, world-wide license to publish the Supplemental Material files. Please contact the corresponding author directly for reuse.
