# Supplementary material for: CalR is an activator of biofilm formation in Vibrio parahaemolyticus
Source: Appl Environ Microbiol. 2025 Sep 11;91(10):e00724-25. doi: 10.1128/aem.00724-25 (PMC12542678; doi:10.1128/aem.00724-25)
Supplement: Supplemental figures — Fig. S1, growth curves of WT and ΔcalR strains; Fig. S2, CalR has no regulatory effect on Op-Tr phase variation. [file aem.00724-25-s0001.docx]

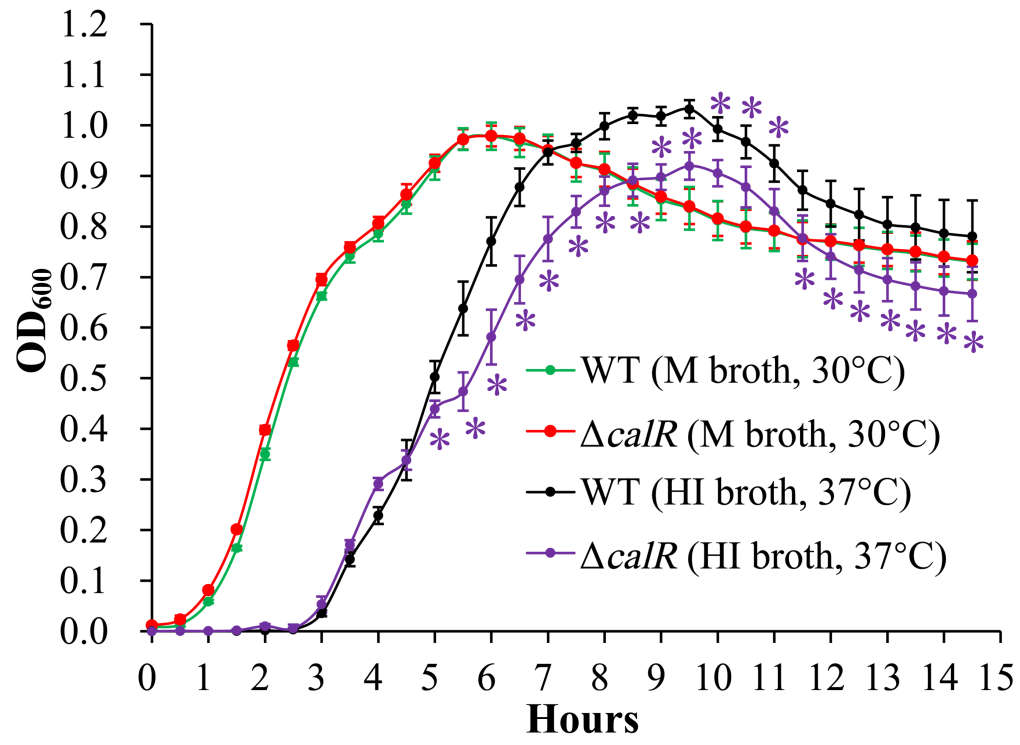


**Fig. S1 Growth curves of WT and *ΔcalR*.** WT and *ΔcalR* were cultured with HI or M broth in a 96-well cell culture plate, and then grown at 37ºC or 30ºC with shaking at 800 rpm in a microbial growth curve analyzer MGC-200. The growth curves were created by monitoring the OD_600_ values of each culture at 30-min intervals. Experiments were performed at least two times with twelve replicates per trial for each condition. The results are expressed as the mean ± SD and analyzed by Paired Student’s *t*-test. *, *P* < 0.05.


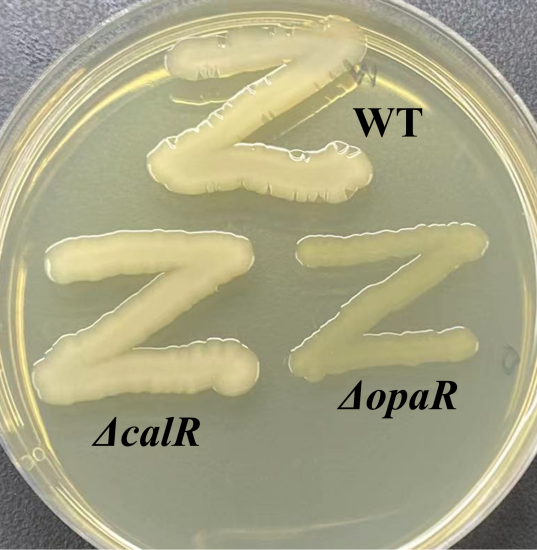


**Fig. S2 CalR has no regulatory effect on Op-Tr phase variation.** A small amount of overnight cell culture of each *V. parahaemolyticus* strain was taken with an inoculation loop, streaked directly on an HI plate, and then statically incubated at 37°C for 24 hours. Pictures are representative of two independent experiments with three replicates each.
