# Supplementary material for: CalR is an activator of biofilm formation in Vibrio parahaemolyticus
Source: Appl Environ Microbiol. 2025 Sep 11;91(10):e00724-25. doi: 10.1128/aem.00724-25 (PMC12542678; doi:10.1128/aem.00724-25)
Supplement: Table S1 — Oligonucleotide primers used in this study. [file aem.00724-25-s0002.docx]

**Table S1. Oligonucleotide primers used in this study**

| **Target** | **Primers (forward/reverse, 5'-3')** | |
| --- | --- | --- |
| **Construction of mutants** | | |
| *calR* | GTAGCTGCAGGCAGATTATTTGACTGATACGC/GTTCGCAAATGGGAAGTCTCTCATCGCATCTTTCTTCTC | |
|  | GAGAAGAAAGATGCGATGAGAGACTTCCCATTTGCGAAC/GTGAGCATGCTACTTACCTTTTGGCTTACAG | |
|  | GTAGCTGCAGGCAGATTATTTGACTGATACGC/GTGAGCATGCTACTTACCTTTTGGCTTACAG | |
| *scrP* | GCGCCTGCAGCTCAGAAGTAGCAATCGTGA / GCAGTTCATCCGTAGTGAGGTACTTTTCTAACGATGCGAC | |
|  | GTCGCATCGTTAGAAAAGTACCTCACTACGGATGAACTGC/GCGTGCATGCACAGGAAACTTACTCTCTCG | |
|  | GCGCCTGCAGCTCAGAAGTAGCAATCGTGA/GCGTGCATGCACAGGAAACTTACTCTCTCG | |
| *vpa0184* | GCGCTCTAGAGTAAATTCCCCCTCAAGTGC/GCTGTCATTGGGCATTAACTCACAAGGTTAGGCACAATGA | |
|  | TCATTGTGCCTAACCTTGTGAGTTAATGCCCAATGACAGC/GCGCGCATGCAGTGATGCCGACAGAAATGC | |
|  | GCGCTCTAGAGTAAATTCCCCCTCAAGTGC/GCGCGCATGCAGTGATGCCGACAGAAATGC | |
| *vpa1429* | GCGCTCTAGAGTTTGCTTTTGTGTATCCTG/TTCGGCTTGAGTTTCTACGCAGTTTTCCCTCTTTGTCG | |
|  | CGACAAAGAGGGAAAACTGCGTAGAAACTCAAGCCGAA/GCGCGCATGCCGAGTGTGAGGTTTCTATTG | |
|  | GCGCTCTAGAGTTTGCTTTTGTGTATCCTG/GCGCGCATGCCGAGTGTGAGGTTTCTATTG | |
| **Construction of complemented mutants** | | |
| *calR* | GCGGTCGACAGGAGGAATTCACCATGTTAGAGAAGAAAGATG/GCGAAGCTTTTATTTTGATGCGACCAC | |
| *vpa1249* | GATCCCGGGAGGAGGAATTCACCATGCAAACTCGCCGCGAA/GACTCTAGATCTCTCATCCGATAAGTCTTG | |
| *scrP* | GATCCCGGGAGGAGGAATTCACCGTGAGAAAGTCGGCTTACGC/GACGTCGACTTAAGACATCAAGTTTTGATCGG | |
| **qPCR** | | |
| *cpsA* | GAGAGCGGCAACCTATATCG/CGCCACGCCAACAGTAATG | |
| *scvE* | GACAGGTCGTGATGCCATTC/GGCGATGATGACCGAAGTG | |
| *scrP* | GCATTCCTGTAATCTCAACC/AGTTCTTCCAAACCAATGGC | |
| *vpa0184* | GCCTATACCGAGTTAGTACG/CATTGGGCATTAACTCGC | |
| *vpa0198* | GCATCAGAATCAGCAAGAC/ATGCTTAGCTCCTCTTCTTC | |
| *vpa1429* | AAGTTGGTCATCGAAAAAG/AAGTGCTGAACAAAATCCG | |
| *16S rRNA* | GACACGGTCCAGACTCCTAC/GGTGCTTCTTCTGTCGCTAAC | |
| **Luminescence assay** | | |
| *cpsA* | GCGCGAGCTCCTTCCCTGTAAATAAGTCATCC/GCGCGGATCCAAGCGAACTCCATCTCATAAG | |
| *scvE* | GCGCGAGCTCAAGAGTCTCGTGAACGGATG/GCGCGGATCCATGGCATCACGACCTGTCTC | |
| *scrP* | | GCGCGAGCTCGGTCTTGCCATTTACGAG/GCGCGGATCCCGCAGCATCGTCTTCCATAC |
| *vpa0184* | | GCGCGAGCTCCAATAGGATGATGAAACCGA/GCGCGGATCCAAGTAAGATTTAGACCCAGC |
| *vpa0198* | | GCGCGAGCTCCTCTGGTTCATTGTCTTG/GCGCGGATCCGTCTTGCTGATTCTGATG |
| *vpa1429* | | GCGCGAGCTCCAATAGGATGATGAAACCGA/GCGCGGATCCAAGTAAGATTTAGACCCAGC |
| **Two-plasmid LacZ fusion assay** | | |
| *cpsA* | | GCGCGTCGACCTTCCCTGTAAATAAGTCATCC/ GCGCGAATTCAAGCGAACTCCATCTCATAAG |
| *scvE* | | GCGCGTCGACAAGAGTCTCGTGAACGGATG/GCGCGAATTCATGGCATCACGACCTGTCTC |
| *scrP* | | GCGCGTCGACGGTCTTGCCATTTACGAG/GCGCGAATTCCGCAGCATCGTCTTCCATAC |
| *vpa0184* | | GCGCGTCGACTCTATGACAACACCTTTAGC/GCGCGAATTCAGCATAGATACAAGAAAACAA |
| *vpa0198* | | GCGCGTCGACCTCTGGTTCATTGTCTTG/GCGCGAATTCGTCTTGCTGATTCTGATG |
| *vpa1429* | | GCGCGTCGACCAATAGGATGATGAAACCGA/GCGCGAATTCAAGTAAGATTTAGACCCAGC |
| **EMSA** | | |
| *cpsA* | GCGCGTCGACCTTCCCTGTAAATAAGTCATCC/GCGCGAATTCAAGCGAACTCCATCTCATAAG | |
| *scvE* | GCGCGTCGACAAGAGTCTCGTGAACGGATG/GCGCGAATTCATGGCATCACGACCTGTCTC | |
| *scrP* | GCGCGTCGACGGTCTTGCCATTTACGAG/GCGCGAATTCCGCAGCATCGTCTTCCATAC | |
| *vpa0184* | GCGCGTCGACTCTATGACAACACCTTTAGC/GCGCGAATTCAGCATAGATACAAGAAAACAA | |
| *vpa0198* | GCGCGTCGACCTCTGGTTCATTGTCTTG/GCGCGAATTCGTCTTGCTGATTCTGATG | |
| *vpa1429* | GCGCGTCGACCAATAGGATGATGAAACCGA/GCGCGAATTCAAGTAAGATTTAGACCCAGC | |
| *toxR* | GCGCGTCGACATCGTTAAGGTATTTGCA/GCGCGAATTCCGAGCGAATTACTATTTGG | |
| 16S rDNA | GACACGGTCCAGACTCCTAC/GTGCTTCTTCTGTCGCTAAC | |
